# Taletrectinib in ROS1-positive NSCLC: bridging clinical trials and real-world practice

**DOI:** 10.1097/MS9.0000000000003945

**Published:** 2025-09-23

**Authors:** Fatima-Tuz Zahra, Fatima Naveed, Mahnoor Fatima

**Affiliations:** a Department of Medicine, Nishtar Medical College, Nishtar Medical University Multan, Pakistan; b Department of Medicine, Shaheed Ziaur Rahman Medical College, Rajshahi Medical University, Bogura, Bangladesh

Lung cancer remains one of the leading causes of cancer-related mortality worldwide, accounting for a significant fraction of the global oncologic burden[1]. In 2020, the incidence of new lung cancer cases was projected to be around 247 270, with 130 340 male and 116 930 female cases. Previous reports have shown that lung cancer accounted for more mortalities alone than breast cancer, colorectal cancer, prostate cancer, and leukemia collectively in males aged over 40 and females aged over 60 years[2]. This article was written in accordance with the TITAN guideline checklist 2025[3].

Non-Small Cell Lung Cancer (NSCLC), a heterogeneous class of tumors, is the most common class of lung cancers, constituting around 80% to 85% of all diagnosed lung cancer cases. NSCLC has a multifactorial origin, including both environmental factors and genetic susceptibility; however, it occurs most commonly among chronic tobacco smokers, making tobacco smoking the major risk factor. Other factors may include asbestos, radon gas exposure, and other environmental and occupational pollutants[1].

However, with significant progress in screening, diagnosis, and treatment, the management of NSCLC has been revolutionized by the emergence of novel advancements in the past decade. The advances in treatment primarily include molecularly targeted therapies, immune checkpoint inhibitors and anti-angiogenic agents, all of which have contributed to markedly improved patient outcomes.

The treatment strategies for NSCLC include surgical resection, chemotherapy, radiotherapy, immunotherapy, and molecularly targeted therapy. The recommended treatment strategy for patients with early-stage NSCLC (stages I, II, and IIIA) is surgical resection, while adjuvant platinum-based chemotherapy is recommended for stages II-IIIA. However, about 30% of patients have locally advanced disease and are non-surgical candidates[4].

For the treatment of advanced NSCLC, molecular targeted therapy has notably improved clinical outcomes. Tyrosine Kinase Inhibitors, targeting oncogenic driver mutations have advanced across multiple generations to improve efficacy and overcome resistance[5].

A revolutionary advancement has been made in the treatment landscape of NSCLC with the recent FDA approval of Taletrectinib, sold under the brand name Ibtrozi. Taletrectinib is a potent, CNS-active, next-generation, highly selective inhibitor of proto-oncogene tyrosine-protein kinase ROS (ROS1) and neurotrophic tyrosine receptor kinase, indicated for the treatment of adult patients with locally advanced or metastatic ROS1-positive NSCLC[6].

Taletrectinib binds to the ATP-binding site of the ROS1 kinase domain, causing inhibition of phosphorylation and downstream signaling pathways, such as MAPK/ERK and PI3K/AKT, which suppresses uncontrolled proliferation of tumor cells.

Unlike the first-generation drugs crizotinib and entrectinib, which are limited by inadequate clinical responses in several patients and the emergence of resistance, especially the ROS1 G2032R mutation, which rendered crizotinib ineffective, taletrectinib was designed to overcome resistance mutations, including G2032R, making it effective where older TKIs fail. Another advantage of taletrectinib over the older generation TKIs is its ability to penetrate the blood-brain barrier and produce strong intracranial activity, controlling brain metastases more efficiently, which was another significant limitation of the older generation TKIs[7].

The phase II regional TRUST- I and global TRUST-II trials were single-arm, open-label, non-randomized, and multicenter studies. The safety population comprised all patients treated with a once-daily dosage of 600 mg taletrectinib pooled across the clinical program^[8,9]^. The TRUST-I trial conducted in China reported remarkable results, with a confirmed ORR (cORR) of 91% and intracranial cORR of 88% in TKI naïve patients, and 52% of cORR and 73% of intracranial cORR in crizotinib-pretreated patients. In TKI-naїve patients, the duration of response (DOR) and progression-free survival (PFS) were not reached at the time of reporting, while in crizotinib-pretreated patients, median PFS approached 8 months, highlighting taletrectinib’s ability to extend disease control even after prior targeted therapy[10]. The global TRUST-II trial confirmed these findings across a broader patient population, demonstrating response rates of over 92% in TKI-naïve patients and about 50% in those patients who were previously treated with a ROS1 inhibitor, with an intracranial response exceeding 90%. Moreover, taletrectinib demonstrated activity against the G2032R resistance mutation, a solvent-front alteration that historically posed a barrier to durable treatment. In TRUST-I, among 13 TKI-pretreated patients with baseline G2032R, 8 (61.5%; 95% CI, 31.6–86.1) achieved an objective response. This highlights taletrectinib’s ability to overcome a crucial barrier in ROS1 + NSCLC[11].

The transition from the trials to the realm of real-world practice highlights the crucial question of how taletrectinib will be deployed outside the controlled environment of TRUST-I and TRUST-II. In clinical practice, brain metastases remain a pivotal treatment challenge in ROS1-positive NSCLC. This has necessitated cranial irradiation, which carries long-term cognitive consequences. Taletrectinib’s high intracranial activity (with response rates of about 77% in TKI-naïve and 66% in crizotinib-pretreated patients) has the potential to delay or even avoid reliance on radiotherapy, thereby showing an improvement in both survival and quality of life. This impact goes beyond efficacy; it may reshape treatment algorithms and lower the long-term neurological morbidity associated with traditional approaches[12].

Taletrectinib has demonstrated a favorable safety profile in ROS1 + NSCLC, with lower discontinuation rates (5.2%) and dose reduction rates (19.1%). Common adverse effects, such as mild transaminase elevation and GI intolerance were quite manageable; and neurological side effects were less frequent compared to entrectinib or repotrectinib^[7,13]^. While TRUST trials showed this safety profile, participants were typically younger and healthier than those in real-world practice. In clinical practice, this drug will be prescribed to older individuals with comorbidities and polypharmacy. In these patients, even mild toxicity poses a far greater risk, underscoring the importance of post-marketing surveillance and registry data to validate taletrectinib’s safety in routine practice.

The efficacy of taletrectinib depends on the timely identification of ROS1 fusions. However, molecular testing remains inconsistent across healthcare systems, particularly in low- and middle-income countries[14]. Without widespread access to next-generation sequencing, many eligible patients might never receive this treatment. Even when patients are accurately identified, the optimal sequencing of ROS1 inhibitors remains an unresolved clinical dilemma. Although taletrectinib has demonstrated efficacy in both first-line and post-crizotinib settings, real-world treatment choices will still be shaped by factors such as drug availability, cost, and local clinical guidelines. In resource-limited systems, crizotinib may continue to serve as the preferred first-line treatment, with taletrectinib reserved for a salvage role. Conversely, in high-income countries, clinicians might increasingly opt for taletrectinib to maximize intracranial disease control and delay resistance.

The greatest obstacle to taletrectinib therapy might be economic. Provided that crizotinib is available at a significantly lower cost, access to taletrectinib might only be restricted to patients in high-income countries or those with comprehensive insurance, exacerbating global disparities in lung cancer care. To address this, proactive strategies are required, ranging from tiered pricing models and generics development to broader compassionate-use programs. These steps are essential to ensure that the advantages of taletrectinib reach a wider population.

Taletrectinib draws attention to the promise and challenges of precision oncology. The approval of this treatment highlights the value of targeted therapy for rare oncogenic subsets; it also emphasizes systemic flaws, such as unequal access to molecular testing, high drug costs, and uncertainty surrounding optimal sequencing. Post-marketing research and real-world registries will be crucial to comprehending how well it works with various patient populations. For ROS1-positive NSCLC, taletrectinib marks a significant breakthrough, providing long-lasting systemic and intracranial control where earlier treatments fell short. Its practical application, however, will rely more on our capacity to guarantee adequate testing, access, and careful incorporation into clinical practice than on molecular potency. The main challenge now is to bridge the gap between trial efficacy and routine care so that patients can benefit from this advancement worldwide.

## Data Availability

Data sharing is not applicable to this article.

## Research Registration Unique Identifying Number (UIN)

None declared.

## Peer and Provenance Statement

Not commissioned, peer review pending.

## Assistance with the study

None declared.
